# Understanding the time period preceding psychiatric hospitalization for moderate to severe suicidal ideation: a qualitative study

**DOI:** 10.1186/s12913-026-14361-0

**Published:** 2026-04-17

**Authors:** Katherine Musacchio Schafer, Julia G. Lebovitz, Olias Muse, Amanda Satterthwaite, Melissa M. Hall, S. Trent Rosenbloom, Peter J. Embi, Colin Walsh

**Affiliations:** 1 Department of Biomedical Informatics, Vanderbilt University Medical Center, Nashville, TN USA; 2 Department of Psychiatry and Behavioral Sciences, Vanderbilt University Medical Center, Nashville, TN USA; 3 Department of Psychology, Vanderbilt University, Nashville, TN USA; 4 Department of Internal Medicine, Vanderbilt University Medical Center, Nashville, TN USA; 5 Department of Pediatrics, Vanderbilt University Medical Center, Nashville, TN USA; 6 Department of Nursing, Vanderbilt University Medical Center, Nashville, TN USA

**Keywords:** Qualitative research, Suicidal ideation, Inpatient psychiatric hospitalization, Psychiatric admission, Psychiatry

## Abstract

**Importance:**

Inpatient psychiatric hospitalizations for suicidal ideation are costly, disruptive, and relatively prevalent occurrences. Psychiatric hospitalizations due to suicidal ideation commonly result from breakdowns in longitudinal outpatient care during which patients lack adequate outpatient care and have a subsequent intensification in their suicidal ideation. In the present project, we seek to understand (a) factors preceding psychiatric hospitalization for suicidal ideation, (b) involvement in and barriers to receiving outpatient mental healthcare, and (c) if patients would have been open to receiving informatics tools delivered via an electronic health record (EHR) embedded patient portal message preceding psychiatric admission for suicidal ideation.

**Objective:**

To explore patients’ experiences leading up to psychiatric hospitalization for suicidal ideation.

**Design, setting, and participants:**

We conducted qualitative interviews with 21 adult patients who were hospitalized due to moderate to severe suicidal ideation and were recruited via purposive sampling. Patients were on a 14-bed psychiatric inpatient unit within a large academic medical center in June of 2025. Data were analyzed via Rapid Qualitative Analysis in July of 2025.

**Main outcome:**

Patients’ experiences preceding psychiatric admission for suicidal ideation.

**Results:**

Subject interviews revealed three major themes. (a) Patients reported escalations in suicidal ideation lasting between one week and three months prior to psychiatric admission. Many reported seeking services at Vanderbilt University Medical Center emergency departments, walk in clinics, and specialty clinics. This is time during which patients could have been identified and connected with outpatient mental healthcare. (b) Most patients were not receiving outpatient mental healthcare but desired it. Costs, wait times, and stigma were significant barriers. (c) Patients were overwhelmingly receptive to the idea that our health system could proactively reach out to them with informatics solutions to overcome barriers to care.

**Conclusions and relevance:**

In the weeks leading up to psychiatric admission due to moderate to severe suicidal ideation, patients’ suicidal ideation intensified. It is possible that patients could have been identified and intervened upon during this time to arrest symptom escalation. Most patients reported that they were not receiving adequate outpatient mental healthcare. Patients reported that they would have been open to Vanderbilt University Medical Center reaching out with a Caring Contact, scheduling an outpatient mental health appointment, or providing a mental health treatment app. Future interventions, including informatics-based solutions to connect patients at high risk of suicidal ideation to outpatient care, will build upon these findings.

**Supplementary Information:**

The online version contains supplementary material available at 10.1186/s12913-026-14361-0.

Psychiatric hospitalizations are not uncommon and account for significant yearly healthcare expenditure, summing to approximately $6 billion per year in the United States (US). Roughly 29% of the US population will be hospitalized for mental illness in their lifetime [1] and every year there are more than 10 million inpatient stays with a principal or secondary diagnosis related to mental and substance use disorders [2]. Many of these hospitalizations are due to high-risk suicidal ideation. One way to conceptualize inpatient psychiatric hospitalizations, particularly those due to suicidal ideation, is by viewing these hospitalizations as being symptomatic of gaps in the health system. Patients experiencing significant psychiatric distress, suicidal ideation for example, fall through gaps in care which allows for symptom exacerbation such that they can no longer be managed on an outpatient basis and must be treated with costly inpatient care [3]. Ideally, healthcare systems would identify patients whose suicidal ideation is intensifying and connect them to outpatient care *before* they necessitate inpatient treatment. In a country that spends $225 billion on mental health treatment annually [4], scalable solutions to reduce inpatient psychiatric hospitalizations could provide wide reaching costs savings to patients, health systems, and the country more broadly [5].

Identification of patients at high risk of suicidal ideation represents one aspect of this challenge, and one that has been relatively widely achieved. Researchers at health systems across the country – for example, Veterans Health Administration, Department of Defense, Kaiser Permanante, Massachusetts General Hospital, Vanderbilt University Medical Center – developed and deployed algorithms that accurately identify patients at high risk of suicidal ideation. These algorithms use routinely-collected data documented in the electronic health record (EHR) – diagnoses, problems list, clinical encounters, nursing documentations – to quantify risk of suicidal ideation and have accuracy as defined as positive predictive values and area under the receiver operator curve that are well within the acceptable ranges. Together this line of research indicated that algorithms can be used at scale to identify patients at high risk of suicidal ideation.

However, the other aspect is connecting these patients to evidence-based interventions. That goal has not yet been achieved, and that is largely because it is yet to be studied. Researchers are yet to determine what patients who have been identified by algorithms as being at high risk of suicidal ideation need or are amenable to in response to this algorithmic identification. Evidence based treatments exist and could be helpful in reducing suicidal ideation. Among these evidenced-based interventions is Caring Contacts [6–9] a low-cost intervention with 40 + years of support. Originally developed in 1976, this validated intervention involves healthcare systems proactively contacting patients who have been identified as being at high risk for suicidality with nothing more than a brief message of support. It requires no additional action from patients or healthcare professionals and a single Caring Contact reduces suicidal ideation by 43%. Likewise, brief outpatient mental health therapy can also reduce the risk of suicidal ideation by up to 40% [10]. However, appointments are often difficult to schedule with patients reporting barriers of long wait times, stigma, cost, and limited outpatient providers. Finally, evidence-based mental health treatment applications also offer scalable low cost interventions that can reduce suicidality [11–14]. Many of these treatment applications improve coping, relaxation, distraction, and positive thinking skills, resulting in as much as a 20% reduction in risk of suicidal ideaiton [15].

To understand what would be helpful in preventing symptom intensification of patients who required inpatient hospitalization due to moderate to severe suicidal ideation and inform just-in-time interventions, we conducted the present qualitative study among adults receiving inpatient psychiatric care at a large academic medical center. The goal of this project was to understand (a) factors that preceded psychiatric hospitalization, (b) involvement in and barriers to receiving outpatient mental healthcare, and (c) if patients would have been open to receiving Caring Contacts, outpatient mental health appointments, evidence based mental health treatment applications via an EHR-enabled patient portal message in the days/weeks preceding psychiatric admission. Findings will inform interventions targeted at reducing suicidal ideation in patients who have been identified by our systemwide algorithm as being at high risk of suicidal ideation.

## Methods

We used qualitative description to focus on patient experiences preceding psychiatric hospitalization due to suicidality. Qualitative description emphasizes describing participant experiences in their language with minimal interpretation and is recommended for research questions that aim to report participant experience. The study was approved by the Institutional Review Board at Vanderbilt University Medical Center. All participants provided written informed consent. This study followed the Consolidated Criteria for Reporting Qualitative Research [16] (COREQ) reporting guideline for qualitative studies. The 32-item checklist is in the Supplemental Files.

The interview guide used in this study was developed for this study and is not published elsewhere. It was developed primarily by the first author, a licensed clinical psychologist with extensive training in qualitative research, implementation science, and quality improvement. The interview guide was then reviewed by masters level research assistants with training in qualitative research methods. It was found to be clear and effective and thus did not require pilot testing. The interview guide is attached in a supplementary file.

### Funding disclosure

This work was supported by Realizing Accelerated Progress, Investigation, Implementation, and Dissemination in Learning Health System (RAPID-LHS) Center is P30HS029767. This P30 Center, supported by the Agency for Healthcare Research and Quality (AHRQ) and PCORI and the Vanderbilt Institute for Clinical and Translational Research (VICTR) is UL1TR002243. This is a Clinical and Translational Science Award (CTSA) from the National Center for Advancing Translational Sciences (NCATS).

### Setting and subjects

Patients were eligible if they were *≥* 18 years of age, experiencing moderate to severe suicidal ideation (i.e., CSSRS of yellow, orange, or red), English speaking, and receiving inpatient psychiatric care at Vanderbilt University Medical Center's Vanderbilt Psychiatric Hospital. We employed purposive sampling to enrich our sample for patients who would benefit from receiving just-in-time outpatient psychiatric care to prevent worsening of suicidal ideation. The study took place in a 14-bed adult psychiatric inpatient unit at an academic medical center between June 02, 2025, and June 23, 2025. All patients on this specific unit are voluntarily admitted to hospitalization. Interviews were conducted in a private space with only the participant and interviewer present. The psychiatric hospital is part of a large health system with seven hospitals and 1,741 beds. Vanderbilt University Medical Center provides over 3 million annual patient visits, 213,000 emergency department visits, and 80,000 hospital discharges. The health system uses Epic EHR software.

### Research team

#### Personal characteristics

In-depth semi-structured interviews were conducted by the first or second author, a licensed clinical psychologist with a Doctor of Philosophy degree (i.e., Ph.D.) and clinical psychology doctoral student with a Master of Science degree (i.e., M.S.), respectively. Both interviewers have CITI, HIPAA, and qualitative methods trainings.

#### Relationship with participants

Interviewers were not known to participants and had no relationships with participants prior to interviews. Participants were informed that researchers were interested in factors preceding psychiatric hospitalization due to suicidal ideation, engagement with outpatient mental healthcare, and use of EHR-enabled patient portal messaging system.

### Study design

#### Data collection

Every morning during the data collection period, the first author reviewed the EHR of all patients on this psychiatric inpatient unit, identifying those who met eligibility criteria. Unit attendings then approved patients stable enough to be approached. Once approved, the first or second author approached each patient and informed them that they were eligible for a study, described the study procedures, and invited them to enroll. Patients who agreed to participate were taken to a private room on the unit were informed consent was obtained. Participants completed a paper document to receive compensation, and a semi-structured interview commenced. Interviews lasted 10 to 30 min. The semi-structured interview guide is in Supplemental File 1 and was tested among Ph.D. level and Masters level researchers with extensive training in qualitative research. No repeat interviews were conducted. Field notes were made during the interviews and data saturation was estimated a priori to emerge at approximately 20 participants. Data saturation was determined in post hoc analyses to be 21 participants. Transcripts were not returned to participants for comment and/or correction.

### Analysis and findings

*Data analysis*. Interviews were digitally audio recorded, responses were documented via pen and paper during the interviews, and files were deidentified. Rapid Qualitative Analysis (RQA) [17] consistent with the qualitative descriptive approach was used to analyze interviews. RQA [17] was performed from June to July of 2025. RQA prioritizes efficiency in extracting themes from in-depth interviews as well as actionable results to implement in practice. It saves time, reduces costs, and improves efficiencies of teams conducting analyses [18]. Participants were compensated $30. Interviews were coded and analyzed by J.L. and K.M.S.

## Results

**Fig. 1:**
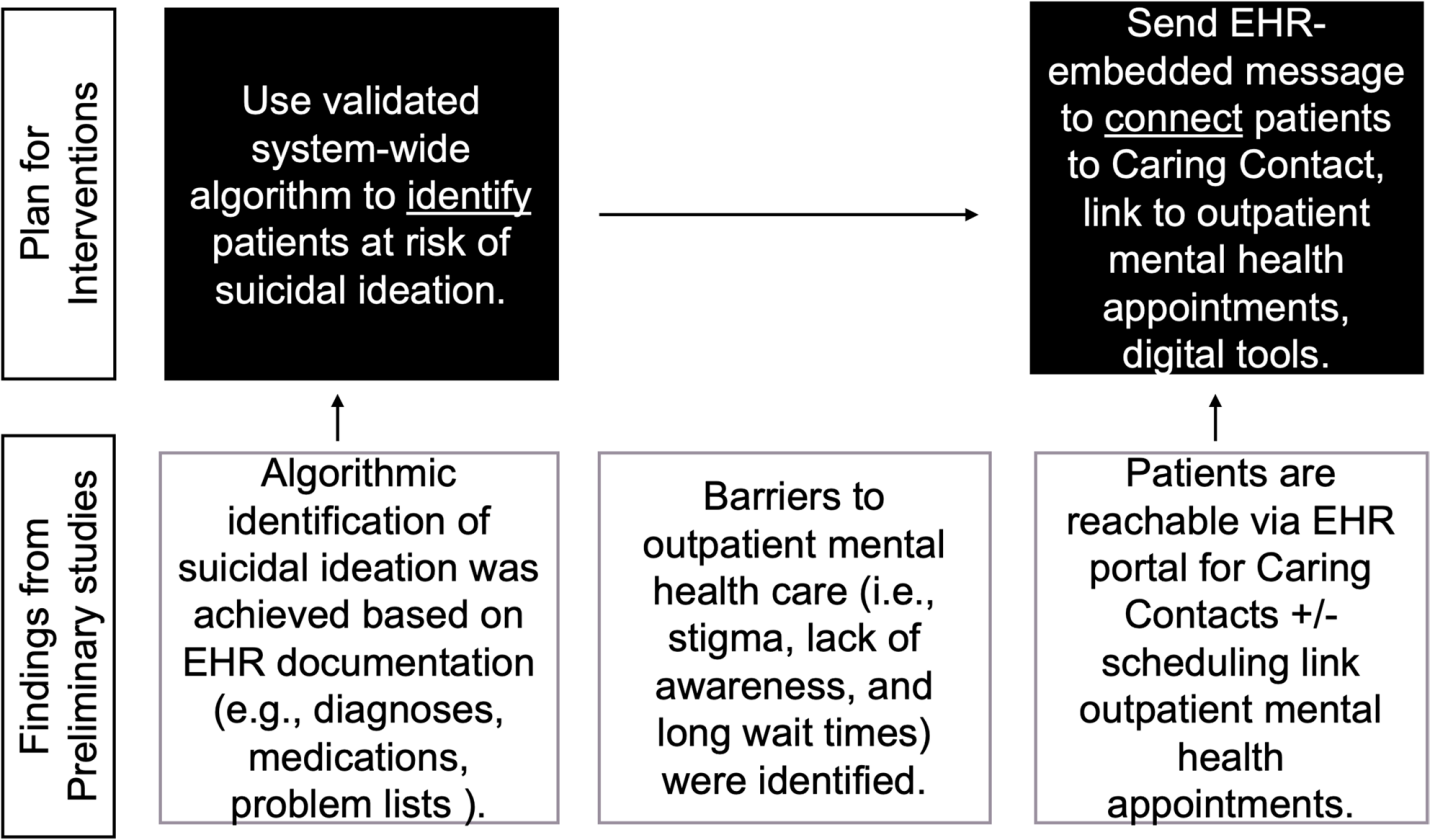
Findings from this project and planned goal for future intervention

Of 23 patients invited to participate, 21 enrolled and were interviewed. One eligible patient was experiencing acute paranoia and shared that they were not comfortable participating in psychological research. Another eligible patient declined to enroll because of lack of interest in the study saying, “It just isn’t my thing”. 21 adults admitted to psychiatric hospitalization for moderate to severe suicidal ideation were enrolled and interviewed (women, *n* = 12; men, *n* = 3; non-binary, *n* = 6; White, *n* = 13, Black/African American, *n* = 7, Asian, *n* = 1; Hispanic or Latino, *n* = 5, Not Hispanic or Latino, *n* = 5). For race, patients could chose more than one value. 18 of the 21 (85.7%) had health insurance at the time of admission. The mean age was 28.85 years (*SD* = 10.33). Three major themes emerged: (a) factors that preceded psychiatric hospitalization, (b) involvement in and barriers to receiving outpatient mental healthcare, and (c) if patients would have been open to receiving Caring Contacts, outpatient mental health appointments, evidence based mental health treatment applications via an EHR-enabled patient portal message in the days/weeks preceding psychiatric admission. Quotes below represent major and minor themes. Quotes were selected on their representativeness of themes that emerged when three or more respondents endorsed similar sentiments. The three major themes are depicted in Fig. 1.

### Major themes

#### Factors preceding hospitalization for suicidal ideation

Patients reported a variety of factors preceding psychiatric hospitalization due to moderate to severe suicidal ideation:

- Patients reported that symptoms intensified in the seven days to three months preceding their inpatient hospitalization. P06 a said “I knew my depression was getting bad a few weeks before I came in. It felt like I was just trying to catch up with myself, but I was so sluggish. I was just trying to hold on and do the bare minimum. And I had been holding on for a while…but eventually I couldn’t hold on anymore.” P13 said “My mental health had been bad for the last three months. My friends and partner could tell and started to have conversations with me about getting help.”
- For many patients, this time was punctuated with care at [redacted for review] affiliated primary care clinics, specialty clinics, and emergency departments. P21 reported that in the seven days preceding inpatient psychiatric hospitalization, she sought care at Vanderbilt University Medical Center’s emergency departments three separate times. P20 reported that in the weeks leading up to psychiatric hospitalization, she saw multiple specialties including endocrinology and primary care. However, some patients were not existing patients within the Vanderbilt University Medical Center system and had never been treated within the [redacted for review] Center prior to psychiatric hospitalization. For example, P16 was being seen by a non-profit mental health cooperative and “didn’t have any doctors at Vanderbilt University Medical Center”. Thus, notes related to encounters external to Vanderbilt University Medical Center would not have been in the Vanderbilt University Medical Center system.

#### Outpatient care

In general patients were either not receiving outpatient mental healthcare or if they were receiving outpatient mental healthcare, it was incomplete (e.g., under the care of a therapist but not a medication provider, receiving therapy but only once every few weeks, receiving psychotropic medications until their medication provider no longer was available). P12 had access to a psychiatrist who she was seeing once a month but was seeing a therapist “once every couple of weeks”. She went on to say about this incomplete outpatient mental health “I am going to be honest, it was just not beneficial over the phone. I didn’t even have video. It was just talking on the phone. And it did not help.” Patients reported that lack of complete outpatient mental healthcare was due to multiple factors:

- Navigating Vanderbilt University Medical Center to obtain outpatient mental services was a barrier. P11 said “I log onto the patient portal. And I just get lost. And then just stop.” This demonstrates both the cumbersome nature of navigating the health system as well as the cognitive load that many patients with psychiatric conditions experience.
- Stigma contributed to insufficient use of outpatient care. P03 said “I just felt like talking to someone wouldn’t help and like I should be able to handle everything on my own. I have always been able to manage without therapy, and I didn’t want to see that things had gotten so bad that I needed to get help.” P16 named this experience very explicitly saying, “my family is very anti-medicine.not very emotion focused. The stigma stopped me from getting help.”
- Long waitlists also contributed lack of outpatient care. P06 said “when things were getting bad, I called many, many providers. Ten, twelve different places. And all of them were completely full. None of them could see me for months. I scheduled an appointment; it is in [three months from now]. And obviously I got hospitalized before then.” Among patients who were receiving outpatient care, they shared difficulty in obtaining a timely appointment with their provider. P18 shared that his doctor was on vacation, and he was unable to be connected with another provider in the group.
- Financial barriers seemed bidirectional in that financial strain likely contributed to the intensification of psychiatric symptoms and made finding treatment more challenging. P10 “I was receiving outpatient therapy, but not consistently because I couldn’t afford it” and “the cost of therapy made it hard for me to see them regularly.” P13 said “finances have been really tight lately and it is super stressful. I can barely afford groceries. Theres no way I can pay for a therapist too.”

#### Open and available to being reached by the system

Most patients said they would have been reachable via the Vanderbilt University Medical Center patient portal messaging system. P11 said “I have been to Vanderbilt University Medical Center many times. I use the patient portal to look at notes after my appointments and to message my doctors.” P05 said “the patient portal is how I talk to my doctors. I have tons of messages on the app right now.” Patients were open to receiving the following content:

- Many patients said they would have benefitted from a Caring Contact. P21 said “if [redacted for review] would have sent me a message of support it would have made me feel hopeful…like I knew that I would be helped.” P14 said “it might have reminded me that there is support. It would have been good to connect me to some resources.” P07 said “I would have felt less isolated.” P09 said “a caring contact would have been encouraging”. However, one patient diverged, saying P03 “I don’t know if a message of support would have made much of a difference.”
- Patients overwhelmingly said it would have been helpful if Vanderbilt University Medical Center] had scheduled them an outpatient appointment. P07 said “oh that would be amazing if Vanderbilt could have scheduled an appointment for me. That was what I wanted. I didn’t want to come inpatient.”
- Participants reported that were open to using a mental health treatment app to cope with symptoms, especially if Vanderbilt had recommended a specific app. P05 “if the app came from Vanderbilt I would have taken that seriously” and “I’ve used other kinds of applications for mental health supporting treatment. Stuff like Finch and Calm. Those are pretty solid. I’ve used those before. But if there were one used that was linked to Vanderbilt that would be awesome.”

### Minor themes

#### Chronic nature of mental health conditions

For many patients, this was not their first psychiatric admission for suicidal ideation. When researchers asked about when mental health symptoms started occurring, multiple patients responded with the same exact response. P11 and P13 both said “Do you mean this time or when did they start in my life?” This indicated that for many patients, mental health concerns had been a chronic, perhaps lifelong condition that they managed to varying degrees. Further, some patients reported that in previous psychiatric crises they received care at Vanderbilt University Medical Center and opted to return to Vanderbilt University Medical Center to get care during this subsequent mental health crisis.

#### Wait times in the psychiatric emergency department

Patients reported wait times in the psychiatric emergency department lasting three to twelve hours. When asked how they felt about this time, patients were generally not bothered by wait times. They were understanding. P16 said “I didn’t find the wait times to be excessive. It was actually shorter than I’d expected.” P15 said “they have a lot to do in the emergency department. They take blood and urine and you talk to a bunch of different doctors.” P01 and P19 felt there was a lack of transparency and communication during this time. P02 shared that during that wait time, they thought “man, I shouldn’t have come in here”.

## Discussion

Psychiatric hospitalizations for suicidal ideation are costly [19], disruptive [3], and common [1, 2]. They can indicate gaps in the health system such that patients deteriorate so much so that they can no longer be treated effectively via outpatient modalities [20]. We present findings from 21 qualitative interviews with adults admitted to a psychiatric hospitalization with suicidality. We sought to uncover (a) the factors that preceded psychiatric hospitalization for suicidal ideation, (b) involvement in and barriers to receiving outpatient mental healthcare, and (c) if patients would have been open to receiving Caring Contacts, outpatient mental health appointments, evidence based mental health treatment applications via an EHR-enabled patient portal message in the days/weeks preceding psychiatric hospitalization.

Three major themes emerged:

1. Patients reported that suicidal ideation intensified in the weeks and months preceding psychiatric. Many indicated that they used the EHR-enabled patient portal to look for outpatient mental healthcare; others said they presented at the emergency department or specialty or primary care services before admission. Evidence of much of this activity can be found in log data via Health IT and could be used to build models to identify patients at high risk of imminent psychiatric hospitalization.
2. In general patients were not engaged in adequate outpatient care and barriers included wait times (e.g., contacting as many as twelve outpatient mental health providers with appointments not available for up to twelve weeks), costs, the cumbersome nature of a large health system, and stigma. This is consistent with the broader literature regarding barriers to outpatient mental healthcare [21–23]. Informatics tools, including an EHR-embedded scheduling tool delivered via the patient portal, could be used to streamline the process of obtaining outpatient mental health appointments.
3. Patients were overwhelmingly available and receptive to being contacted by [redacted for review] during that time of distress preceding psychiatric hospitalization for suicidal ideation. They would have appreciated a Caring Contact, scheduled outpatient mental health appointment, or [redacted for review] verified treatment app.

Algorithms could be used to improve healthcare delivery. EHR log data including documentation from primary and specialty clinics as well as emergency department visits could be used to identify patients at risk of psychiatric hospitalization due to suicidal ideation. Patient portal messaging (including a Caring Contacts, a scheduling tool for outpatient mental health, and a mental health treatment app) could connect patients to outpatient mental healthcare, potentially reducing the risk that their suicidal ideation would intensify such that it would require care on an inpatient basis.

It is important to note that on their own, these interventions are unlikely to reduce suicidal ideation, connect patients to outpatient mental healthcare, or reduce the costly inpatient healthcare utilization that is associated with episodes intense suicidal ideation. For example, Caring Contacts on its own has been found to reduce suicidal ideation however those messages are sent over a series of weeks and would likely not assuage suicidal ideation or connect patients to outpatient mental healthcare immediately when they need it while in crisis. Likewise, a mental health treatment app – even those with strong evidence of therapeutic benefit – would likely be bolstered by parallel outpatient mental health appointments. Thus, with this in mind, we recommend that interventions targeted at reducing suicidal ideation and the associated inpatient care among outpatients who have been identified by an algorithm as being at high risk of suicidal ideation use a dual approach, combining evidence-based treatments. By combining evidence-based treatments, patients will likely experience faster and more enduring reduction in their suicidal ideation.

## Limitations

Limitations relate primarily to the generalizability of these findings. Primarily, these findings are reflective of a sample that was collected based the presence of moderate to severe suicidal ideation. These findings might not generalize to other psychiatric populations, including those presenting to inpatient care for substance use, psychosis spectrum, or personality disorders. Likewise, these findings may not generalize to health systems caring for different populations (e.g., Veterans connected with VA or Service Members connected with DOD healthcare). Indeed systems caring for divergent populations may find their patients exhibit different behaviors precede psychiatric hospitalization for suicidal ideation. This means that algorithmic prediction of – and preventive interventions for – imminent psychiatric hospitalization for suicidal ideation would vary between health systems and algorithms might not seamlessly transfer between health systems. Similarly, these findings would likely not generalize to patients who are new to health systems. Identifying patients based on EHR log data is only feasible among patients who are existing Vanderbilt University Medical Center patients. People who are not existing Vanderbilt University Medical Center patients would not have an EHR chart to find documentation of care in primary or specialty clinics, emergency departments, or patient portal messages to use in algorithmic prediction of psychiatric hospitalization for suicidal ideation. Further, proactively contacting these patients would be impossible as they would not have an EHR-enabled patient portal connected with Vanderbilt University Medical Center. Finally, this entire mode of care, wherein a patient is identified as high risk of having suicidal ideation using data found in, contacted through, and scheduled within EHR patient portal relies on patients having and accessing an EHR-embedded patient portal. However, many patients may not have the resources or knowledge of this patient portal; thus, they would be unreachable via this type of intervention. As such, researchers should investigate other modes of interventions through which to contact these patients, perhaps through text messages, phone calls, or postcards.

## Conclusion

Adults with moderate to severe suicidal ideation receiving care at an inpatient psychiatric unit were interviewed about experiences preceding admission. Three themes emerged. (1) In the weeks and months preceding admission, patients’ suicidal ideation intensified. Many patients sought care at Vanderbilt University Medical Center walk in clinics, emergency departments, and primary and specialty care clinics. Patients also attempted to obtain outpatient mental healthcare, often to no avail. Much of this activity could be found in EHR log data and used to build a model identifying patients at risk of psychiatric admission. (2) Most patients reported that they were not receiving adequate outpatient mental healthcare. Barriers included prohibitive costs, wait times, stigma, and the ability to navigate large health systems. (3) Patients reported that they would have been amenable to and reachable for Vanderbilt University Medical Center reaching out with a Caring Contact, scheduling an outpatient mental health appointment, or providing a mental health treatment app. These findings will be used to inform interventions, including interventions to connect patients at imminent risk of psychiatric hospitalizations for suicidal ideation to supportive messages, outpatient care, and digital therapeutics, potentially averting the need for inpatient psychiatric admission secondary to suicidal ideation.

## Supplementary Information

Below is the link to the electronic supplementary material.

Supplementary Material 1

## Data Availability

Data are available on request due to privacy/ethical restrictions. To submit requests for data, please contact Dr. Katherine Musacchio Schafer at Vanderbilt University Medical Center, Department of Biomedical Informatics, Katherine.m.schafer@vumc.org.

## References

[1] Pedersen CB, Mors O, Bertelsen A, et al. A Comprehensive Nationwide Study of the Incidence Rate and Lifetime Risk for Treated Mental Disorders. JAMA Psychiatry. 2014;71(5):573–81. 10.1001/jamapsychiatry.2014.16. 24806211 10.1001/jamapsychiatry.2014.16

[2] Smith MW, Stocks C, Santora PB. Hospital Readmission Rates and Emergency Department Visits for Mental Health and Substance Abuse Conditions. Community Ment Health J. 2015;51(2):190–7. 10.1007/s10597-014-9784-x. 25563483 10.1007/s10597-014-9784-x

[3] Pathare S, Brazinova A, Levav I. Care gap: a comprehensive measure to quantify unmet needs in mental health. Epidemiol Psychiatric Sci. 2018;27(5):463–7. 10.1017/S2045796018000100. 10.1017/S2045796018000100 PMC6999014 29521609

[4] Mark TL, Levit KR, Yee T, Chow CM. Spending On Mental And Substance Use Disorders Projected To Grow More Slowly Than All Health Spending Through 2020. Health Aff. 2014;33(8):1407–15. 10.1377/hlthaff.2014.0163. 10.1377/hlthaff.2014.0163 25092843

[5] Tibirna A, Petrescu C, Ciobanu CA, et al. Hospitalization costs and mental health: challenges and solutions from recent research - a narrative review. Public Health Healthcare. Preprint 2024 Jun 20. 10.20944/preprints202406.1341.v1.

[6] Landes SJ, Kirchner JE, Areno JP, et al. Adapting and implementing Caring Contacts in a Department of Veterans Affairs emergency department: a pilot study protocol. Pilot Feasibility Stud. 2019;5(1):115. 10.1186/s40814-019-0503-9. 31624637 10.1186/s40814-019-0503-9 PMC6785900

[7] Radin AK, Shaw J, Brown SP, et al. Comparative effectiveness of two versions of a caring contacts intervention in healthcare providers, staff, and patients for reducing loneliness and mental distress: A randomized controlled trial. J Affect Disord. 2023;331:442–51. 10.1016/j.jad.2023.03.029. 36963515 10.1016/j.jad.2023.03.029 PMC10304492

[8] Reger MA, Luxton DD, Tucker RP, et al. Implementation methods for the caring contacts suicide prevention intervention. Prof Psychology: Res Pract. 2017;48(5):369–77. 10.1037/pro0000134.

[9] Skopp NA, Smolenski DJ, Bush NE, et al. Caring contacts for suicide prevention: A systematic review and meta-analysis. Psychol Serv. 2023;20(1):74–83. 10.1037/ser0000645. 35420858 10.1037/ser0000645

[10] Cook JA, Burke-Miller JK, Razzano LA, Steigman PJ, Jonikas JA, Santos A. Serious mental illness, other mental health disorders, and outpatient health care as predictors of 30-day readmissions following medical hospitalization. Gen Hosp Psychiatry. 2021;70:10–7. 10.1016/j.genhosppsych.2021.02.004. 33639449 10.1016/j.genhosppsych.2021.02.004

[11] Bush NE, Dobscha SK, Crumpton R, et al. A Virtual Hope Box Smartphone App as an Accessory to Therapy: Proof-of-Concept in a Clinical Sample of Veterans. Suicide Life-Threatening Behav. 2015;45(1):1–9. 10.1111/sltb.12103. 10.1111/sltb.12103 24828126

[12] Gerner JL, Tucker RP, Moscardini EH, Bagge CL, Reger MA. The Virtual Hope Box mobile application: A systematic review of the literature. Suicide Life-Threatening Behav. 2024;54(3):501–14. 10.1111/sltb.13061. 10.1111/sltb.13061 38380558

[13] Moscardini EH, Le TP, Cowan T, et al. Frequency and predictors of virtual hope box use in individuals experiencing suicidal ideation: An ecological momentary assessment investigation. Suicide Life-Threatening Behav. 2024;54(1):61–9. 10.1111/sltb.13018. 10.1111/sltb.13018 PMC10922839 37960986

[14] A Virtual Hope Box: Randomized Controlled Trial of a Smartphone App for Emotional Regulation and Coping With Distress | Psychiatric Services. Accessed March 31. 2025. https://psychiatryonline.org/doi/full/10.1176/appi.ps.201600283 10.1176/appi.ps.201600283 27842473

[15] Denneson LM, Smolenski,Derek J, Bauer, Brian W, Dobscha, Steven K, Bush NE. The Mediating Role of Coping Self-Efficacy in Hope Box Use and Suicidal Ideation Severity. Archives Suicide Res. 2019;23(2):234–46. 10.1080/13811118.2018.1456383. 10.1080/13811118.2018.1456383 29624123

[16] Consolidated criteria for reporting qualitative research (COREQ): a 32-item checklist for interviews and focus groups | International Journal for Quality in Health Care | Oxford Academic. Accessed June 12. 2025. https://academic.oup.com/intqhc/article/19/6/349/1791966 10.1093/intqhc/mzm042 17872937

[17] Keniston A, McBeth L, Astik G, et al. Practical Applications of Rapid Qualitative Analysis for Operations, Quality Improvement, and Research in Dynamically Changing Hospital Environments. Joint Comm J Qual Patient Saf. 2023;49(2):98–104. 10.1016/j.jcjq.2022.11.003. 10.1016/j.jcjq.2022.11.003 36585315

[18] Lewinski AA, Crowley MJ, Miller C, et al. Applied Rapid Qualitative Analysis to Develop a Contextually Appropriate Intervention and Increase the Likelihood of Uptake. Med Care. 2021;59:S242. 10.1097/MLR.0000000000001553. 33976073 10.1097/MLR.0000000000001553 PMC8132894

[19] Cryer L, Shannon SB, Van Amsterdam M, Leff B. Costs For ‘Hospital At Home’ Patients Were 19% Lower, With Equal Or Better Outcomes Compared To Similar Inpatients. Health Aff. 2012;31(6):1237–43. 10.1377/hlthaff.2011.1132. 10.1377/hlthaff.2011.1132 22665835

[20] Cases C, Lafont Rapnouil S, Gallini A, Arbus C, Salles J. Evidence of practice gaps in emergency psychiatric care for borderline personality disorder: how can this be explained? BMC Psychiatry. 2020;20(1):476. 10.1186/s12888-020-02892-7. 32993589 10.1186/s12888-020-02892-7 PMC7526189

[21] Andrade LH, Alonso J, Mneimneh Z, et al. Barriers to mental health treatment: results from the WHO World Mental Health surveys. Psychol Med. 2014;44(6):1303–17. 10.1017/S0033291713001943. 23931656 10.1017/S0033291713001943 PMC4100460

[22] Carbonell Á, Navarro-Pérez JJ, Mestre MV. Challenges and barriers in mental healthcare systems and their impact on the family: A systematic integrative review. Health Soc Care Commun. 2020;28(5):1366–79. 10.1111/hsc.12968. 10.1111/hsc.12968 32115797

[23] Haugen PT, McCrillis AM, Smid GE, Nijdam MJ. Mental health stigma and barriers to mental health care for first responders: A systematic review and meta-analysis. J Psychiatr Res. 2017;94:218–29. 10.1016/j.jpsychires.2017.08.001. 28800529 10.1016/j.jpsychires.2017.08.001

