# Supplementary material for: Understanding the time period preceding psychiatric hospitalization for moderate to severe suicidal ideation: a qualitative study
Source: BMC Health Serv Res. 2026 Apr 17;26:767. doi: 10.1186/s12913-026-14361-0 (PMC13217970; doi:10.1186/s12913-026-14361-0)
Supplement: Supplementary file 1 — Supplementary Material 1 [file 12913_2026_14361_MOESM1_ESM.docx]

Interview Looking at MHAV Message to Prevent Inpatient Psychiatric Stays

Thanks for taking the time to meet with me. In this study we are seeking input from patients with suicidality who are receiving services from Vanderbilt University Medical Center (VUMC). We want to learn from patients about what contributed to their decision to become connected to mental health resources. You will be asked questions about the decision-making process that preceded your hospital admission. This interview will be a chance for you to share about your experiences and will last about 30 minutes. You will be compensated for this time, at $30. There aren’t any risks to you in participating in this study. You may stop at any time. And none of this information will be entered into your electronic medical record or shared with your treatment team.

When did you start to realize that your mental health symptoms were becoming unbearable and that you might need to be hospitalized? (Days? Weeks?)

And when did you come onto the unit?

During the time that you were struggling would you have been reachable via My Health At Vanderbilt?

How would a Caring Contact (a message of support from Vanderbilt) have affected you during that time you were struggling?

Would a Caring Contact made you feel better during that time?

Would you have been open to receiving a mental health phone app during that time?

This would be an app you could download and use on your phone to help you cope during stressful times.

Would you have been open to Vanderbilt scheduling a mental health appointment for you during that time that your symptoms were becoming unbearable?

Were you receiving outpatient care?

Therapy? (Psychologist? Counselor? Social worker?)

Med provider? (Psychiatrist? Nurse practitioner?)

Did you want to have an outpatient appointment?

What made it difficult for you to schedule an outpatient appointment during that time?

How long did you wait in the emergency department or in the psychiatric assessment services before you came up to the unit?

Did you find that wait time to be excessive?

How could that time have been better used?

What do you think we should study? Focus on?

What would you like to know about getting connected to mental health resources?

Is there anything else you would like to tell us about your decision to come into inpatient or the time leading up to it?
